# Serum vitamin D levels and survival of patients with colorectal cancer: Post-hoc analysis of a prospective cohort study

**DOI:** 10.1186/1471-2407-10-347

**Published:** 2010-07-02

**Authors:** Hidetoshi Mezawa, Tsutomu Sugiura, Michiaki Watanabe, Chihiro Norizoe, Daisuke Takahashi, Akira Shimojima, Seryna Tamez, Yusuke Tsutsumi, Katsuhiko Yanaga, Mitsuyoshi Urashima

**Affiliations:** 1 Division of Molecular Epidemiology, Jikei University School of Medicine, Nishi-shimbashi, Minato-ku, Tokyo, Japan; 2 Department of Paediatrics, Jikei University School of Medicine, Nishi-shimbashi, Minato-ku, Tokyo, Japan; 3 Department of Surgery, Jikei University School of Medicine, Nishi-shimbashi, Minato-ku, Tokyo, Japan

## Abstract

**Background:**

Recently, serum 25-hydroxyvitamin D (25OHD) levels were shown to be associated with the survival of patients with colorectal cancer. However, 25OHD levels were measured a median of 6 years before diagnosis or were predicted levels. In this study, we directly measured serum 25OHD levels at surgery and examined the association with survival among patients with colorectal cancer.

**Methods:**

We started a prospective cohort study to find prognostic factors in patients with colorectal cancer from 2003 to 2008 and stored serum samples and clinical data. As part of a post-hoc analysis, serum 25OHD levels were measured by radioimmunoassay. Association between overall survival and serum 25OHD levels were computed using the Cox proportional hazard model adjusted for month of serum sampling as well as age at diagnosis, gender, cancer stage, residual tumor after surgery, time period of surgery, location of tumor, adjuvant chemotherapy and number of lymph nodes with metastasis at surgery. Unadjusted and adjusted hazard ratios (HR) and 95% confidence intervals (95% CI) were determined.

**Results:**

Serum 25OHD levels were measured in 257 patients. Only 3% had sufficient levels (30 ng/ml and greater). Based on month of blood sampling, an annual oscillation of 25OHD levels was seen, with levels being lower in spring and higher in late summer. Higher 25OHD levels were associated with better overall survival under multi-variate analysis (HR, 0.91: 95% CI, 0.84 to 0.99, *P *= 0.027).

**Conclusions:**

These results suggest that higher 25OHD levels at surgery may be associated with a better survival rate of patients with colorectal cancer.

## Background

Sunlight exposure has been suggested to reduce cancer risk [1]. In addition, living at higher latitudes with lower sunlight exposure is positively associated with cancer mortality [2]. Because vitamin D is made under the skin by exposure to ultraviolet-B radiation in sunlight, low levels of serum vitamin D may contribute to a higher risk of morbidity and mortality associated with colon cancer [3]. One plausible explanation for why increased sun exposure and higher circulating levels of vitamin D are associated with a decreased risk of deadly cancers is that epithelial cells convert the primary circulating form of vitamin D, 25-hydroxyvitamin D (25OHD), to its active form, 1,25-dihydoroxyvitamin D, inside the cells; this active form binds to vitamin D receptors in the nucleus to regulate a variety of genes [4]. These genes help prevent malignant transformation by keeping cellular proliferation and differentiation within normal ranges. In turn, if a cell becomes malignant, 1,25-dihydroxyvitamin D can induce apoptosis and prevent angiogenesis, thereby reducing the potential for the malignant cell to survive.

Two meta-analyses showed that vitamin D deficiency is a risk factor for the development of colorectal cancer [5,6]. Sporadic colon cancer has been induced by a western diet in a mouse model, and was prevented by increasing dietary calcium and vitamin D levels [7]. A pilot randomized, double-blind, placebo-controlled clinical trial showed that vitamin D reduced cell proliferation and increased BCL2-associated X protein, an apoptosis promoter, in colorectal mucosa [8-10]. High doses (1,100 IU) of vitamin D plus calcium were shown to significantly reduce cancer incidence in women [11], although low doses (400 IU) of vitamin D did not decrease the incidence of colorectal cancer [12]. Recently, Ng et al. demonstrated that higher pre-diagnosis blood 25OHD levels were associated with a significant improvement in overall survival of patients with colorectal cancer [13]. However, they only had a single measurement of plasma 25OHD levels taken a median of 6 years before diagnosis. Next, they calculated post-diagnosis 25OHD levels using race, geographic region, and baseline values of physical activity, body mass index, and vitamin D intake reported 1 to 4 years after colorectal cancer diagnosis according to Giovannucci's method [14]. Using these predicted 25OHD levels, they demonstrated that higher 25OHD levels after diagnosis of colorectal cancer may be associated with improved survival [15]. For a more accurate portrayal of 25OHD levels, we collected blood samples at surgery, measured 25OHD levels directly, and investigated the relationship between individual serum levels of 25OHD and overall survival in patients diagnosed with colorectal cancer, according to the vitamin D hypothesis [3].

## Methods

### Informed consent

This study was designed as post-hoc analysis of a prospective cohort study to find prognostic markers in the serum of patients with colorectal cancer from May 2003 to January 2008 and approved by the Ethics Committee for Biomedical Research of the Jikei Institutional Review Board, Jikei University School of Medicine, Tokyo, Japan. All patients provided written informed consent.

### Study Population

Peripheral blood samples were obtained from colorectal cancer patients who underwent surgery at the Department of Surgery, Jikei University Hospital. Patients who were treated with chemotherapy and/or radiation before surgery to reduce the size of the tumor were excluded. Two hundred and fifty-seven patients were included in this study. Prognostic factors known to influence colorectal cancer mortality were extracted from the medical record, including age at surgery, tumor stage, primary tumor location, and year of diagnosis. According to the tumor-node-metastasis system of the American Joint Committee on Cancer [16], stages (I, II, III and IV) were determined based on pathologic analysis of the surgical specimens [17]. Residual tumor after surgery was classified into three categories: R0, no residual tumor; R1, microscopic residual tumor; and R2, macroscopic residual tumor [18]. Metastases to lymph nodes resected at surgery were pathologically examined and counted. All patients were periodically (every 0.5 to 2 months) examined on an outpatient basis to make sure they had not relapsed. Examinations consisted of standard tests, including colonoscopy and computed tomography. Patients were followed until July 31, 2009, or death, whichever came first. Because serum 25OHD levels were higher in the first half of the study span, the time period of surgery was divided into two groups (between May 2003 and December 2005 and between January 2006 and January 2008) and was used as one of the covariates.

### Samples and 25OHD measures

Pathological stages and number of lymph nodes with metastasis were determined the same day or the next day after surgery. In each case, serum samples were obtained in the peri-operative period and stored at -80°C until 25OHD was measured. Serum 25OHD levels were measured twice by radioimmunoassay at SRL Inc. (Hachioji, Tokyo, Japan), as described previously [19,20]. When duplicated data differed by 5 ng/mL or more, measures were repeated, although this occurred in only 7 cases. Personnel who measured 25OHD levels were blinded to clinical information.

### Endpoints

Because some patients could not obtain complete remission after surgery, death was used as the primary endpoint in all patients, and relapse was used as the secondary endpoint in patients who could obtain complete resection after surgery.

### Statistical analysis

Analysis of variance and chi-square test were used to evaluate differences in patient characteristics between 25OHD levels, which were divided into quartiles (3-7 ng/mL, 8-10 ng/mL, 11-15 ng/mL, and 16-36 ng/mL). Overall survival, cancer-specific survival, and disease-free survival curves were compared with the serum 25OHD levels using Cox proportional hazard models with or without multivariate analysis using age at diagnosis (years old), gender, calendar month of blood sampling, cancer stage (I, II, III, and IV), residual tumor (R0, R1, and R2), number of lymph nodes with metastasis, and time period of surgery (between May 2003 and December 2005 and between January 2006 and January 2008). Adjusted hazard ratios (HR) and 95% confidence intervals (CI) were computed. All statistical analyses were performed using STATA 9.1 (STATA Corp., College Station, TX). *P *< 0.05 was considered statistically significant.

## Results

### Serum 25OHD levels

**Figure 1:**
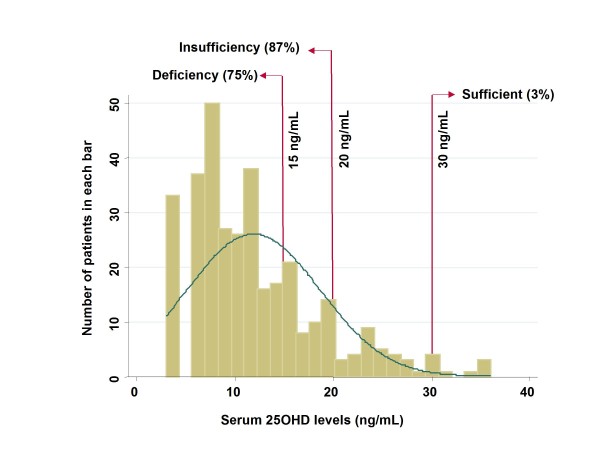
**Histogram of serum 25OHD levels**.

Serum 25OHD levels were measured in 257 patients (Fig. 1). Most patients (85%) showed insufficient levels (less than 20 ng/mL) of 25OHD; only 3% had sufficient levels (30 ng/ml and greater). Quartile points of distribution were as follows: 25%, 7 ng/mL; 50%, 10 ng/mL; and 75%, 15 ng/mL.

**Figure 2:**
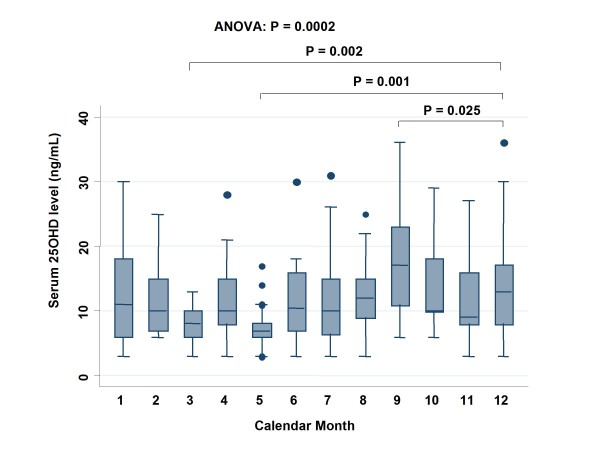
**Serum 25OHD levels in each calendar month**. All points outside the range represented by circles are considered outliers.

Levels of 25OHD were compared based on month of blood sampling (Fig. 2). There was an annual oscillation in 25OHD levels, which were lower in spring and higher in late summer. Compared with December, 25OHD levels were significantly lower in March (*P *= 0.002) and May (*P *= 0.001) and significantly higher in September (*P *= 0.025).

Patients' characteristics according to quartiles of 25OHD levels are shown in Table 1. There were no significant differences in quartiles between age at surgery, gender, cancer stage, location of the tumor, and number of lymph nodes with metastasis. On the other hand, an earlier time period of surgery was associated with significantly higher (*P *< 0.001) 25OHD levels than a later surgical time period.

**Table 1:** Patient characteristics according to quartile of serum 25OHD (n = 257)

	Quartile 1 (3-7 ng/mL) n = 71	Quartile 2 (8-10 ng/mL) n = 58	Quartile 3 (11-15 ng/mL) n = 65	Quartile 4 (16-36 ng/mL) n = 63	*P *value
Serum 25OHD, ng/mL, mean ± SD	5.2 ± 1.7	9.0 ± 0.8	12.6 ± 1.4	21.9 ± 5.2	
Age at surgery, years	64 ± 15	66 ± 13	65 ± 12	65 ± 10	0.76*^1^
Gender, % female	41	43	34	26	0.18*^2^
Stage, %					0.78*^2^
Stage I	21	17	18	24	
Stage II	23	31	37	22	
Stage III	35	32	31	35	
Stage IV	21	20	14	19	
Location of tumor,%					0.24*^2^
Proximal colon	38	44	40	27	
Distal colon	32	19	18	23	
Rectum	30	37	42	50	
Type of resection, %					0.84*^2^
R0	76	79	82	81	
R1	9	10	8	4	
R2	15	11	10	15	
Number of lymph nodes with metastasis	2.2 ± 4.0	3.0 ± 6.8	2.2 ± 4.0	1.6 ± 3.0	0.87*^3^
Time period of surgery					< 0.001*^2^
May 2003 - December 2005	27	43	55	65	
January 2006 - January 2008	73	57	45	35	

*1: *P *value was calculated using one-way analysis of variance.

*2: *P *value was calculated using chi-square test.

*3: P value was calculated using Kruskal-Wallis rank test.

R0, no residual tumor; R1, microscopic residual tumor; R2, macroscopic residual tumor.

### Overall survival and 25OHD

Among 257 patients with colorectal cancer, there were 39 deaths, 30 of which were colorectal cancer-specific deaths. The median time of follow-up of participants still alive at the end of the study was 32.4 months. We assessed the influence of serum 25OHD levels on overall survival of patients using the Cox proportional hazard model adjusted for age at diagnosis (years old), gender, calendar month of blood sampling, cancer stage (I, II, III, and IV), assessment of residual tumor after surgery (R0, R1, and R2), period of surgery, and number of lymph nodes with metastasis (Table 2). Higher serum 25OHD levels were associated with a significant reduction in the risk of overall survival with adjustment for age at diagnosis, gender, calendar month of blood sampling, cancer stage, residual tumor after surgery, time period of surgery, location of tumor, adjuvant chemotherapy, and number of lymph nodes with metastasis (HR, 0.91: 95%CI, 0.84 to 0.99, *P *= 0.027). When we divided 25OHD into quartiles and computed overall survival using the Cox proportional hazard model, the highest 25OHD quartile was associated with a significant reduction in death rate with adjustment for the same factors (HR, 0.16: 95% CI, 0.04 to 0.63, *P *= 0.009) (Table 3). Without adjustment, there was no association between 25OHD quartiles and overall survival. There was no significant association between 25OHD levels and colorectal cancer-specific death (Table 4) or disease-free survival (Table 5).

**Table 2:** Cox proportional hazard models of overall survival without or with multivariate adjustment*^1^

Variable	Single-variate analyses			Multivariate analysis		
		
	Crude HR	95% CI	*P *value	AHR	95% CI	*P *value
25OHD, ng/mL	0.98	0.93-1.02	0.31	0.91	0.84-0.99	0.027
Age at surgery, years	1.01	0.98-1.04	0.49	1.07	1.03-1.12	0.002
Gender, female	1.26	0.66-2.41	0.49	1.59	0.49-5.15	0.44
Time period of surgery						
May 2003 - December 2005	0.94	0.48-1.85	0.87	0.41	0.13-1.27	0.12
January 2006 - January 2008	reference					
Stage						
Stage I	0.00	-	-	0.00	-	-
Stage II	0.02	0.01-4.32	< 0.001	0.02	0.02-1.74	0.13
Stage III	0.13	0.07-0.27	< 0.001	1.13	0.16-7.85	0.90
Stage IV	Reference			Reference		
Location of tumor						
Proximal colon	1.07	0.52-2.22	0.86	1.42	0.55-3.68	0.47
Distal colon	0.45	0.15-1.34	0.15	0.42	0.09-1.94	0.27
Rectum	Reference			Reference		
Type of resection, %						
R0	0.04	0.02-0.09	< 0.001	0.01	0.00-0.11	< 0.001
R1	1.04	0.48-2.26	0.92	0.44	0.11-1.78	0.25
R2	Reference			Reference		
Adjuvant chemotherapy	6.06	2.15-17.10	0.001	0.75	0.17-3.34	0.71
Number of lymph nodes with metastasis*^2^	1.08	1.05-1.11	< 0.001	1.09	1.02-1.16	0.016

*1: Multivariate hazard ratios (HR), 95% confidence intervals (CI), and *P *values were adjusted for age at diagnosis (years), gender, calendar month of blood sampling, cancer stage (I, II, III, and IV), residual tumor after surgery (R0, no residual tumor; R1, microscopic residual tumor; R2, macroscopic residual tumor), time period of surgery (between May 2003 and December 2005 or between January 2006 and January 2008), location of tumor, adjuvant chemotherapy, and number of lymph nodes with metastasis.

*2: Lymph nodes were resected at surgery, and metastases in lymph nodes were confirmed pathologically.

**Table 3:** Cox proportional hazard models with multivariate adjustment*^1^

Covariate	Hazard Ratio	*P *value	95% Confidence Interval
25OHD, ng/mL			
3-7 (Quartile 1)	0.50	0.22	0.16-1.54
8-10 (Quartile 2)	0.55	0.29	0.18-1.65
11-15 (Quartile 3)	Reference		
16-36 (Quartile 4)	0.16	0.009	0.04-0.63

*1: Multivariate hazard ratios, 95% confidence intervals, and *P *values were adjusted for age at diagnosis (years), gender, calendar month of blood sampling, cancer stage (I, II, III, and IV), residual tumor after surgery (R0, no residual tumor; R1, microscopic residual tumor; R2, macroscopic residual tumor), time period of surgery (between 2003 and 2005 or between 2006 and 2008), and number of lymph nodes with metastasis.

*2: Lymph nodes were resected at surgery and metastases in lymph nodes were confirmed pathologically.

**Table 4:** Cox proportional hazard models of cancer death without or with multivariate adjustment*^1^

Variable	Single-variate analyses			Multivariate analysis		
		
	Crude HR	95% CI	*P *value	AHR	95% CI	*P *value
25OHD, ng/mL	0.99	0.94-1.05	0.75	0.98	0.89-1.08	0.67
Age at surgery, years	0.98	0.96-1.01	0.26	1.02	0.96-1.08	0.57
Gender, female	1.36	0.65-2.85	0.41	1.14	0.24-5.34	0.87
Time period of surgery						
May 2003 - December 2005	1.45	0.67-3.13	0.87	1.32	0.28-6.19	0.73
January 2006 - January 2008	reference					
Stage						
Stage I	0.00	-	-	0.00	-	-
Stage II	0.00	-	< 0.001	0.00	-	-
Stage III	0.01	0.04-0.20	< 0.001	0.49	0.02-10.28	0.65
Stage IV	Reference			Reference		
Location of tumor						
Proximal colon	0.77	0.31-1.88	0.56	0.66	0.17-2.56	0.54
Distal colon	0.56	0.18-1.74	0.32	0.36	0.05-2.44	0.30
Rectum	Reference			Reference		
Type of resection, %						
R0	0.03	0.01-0.07	< 0.001	0.02	0.00-0.75	0.034
R1	0.94	0.40-2.18	0.88	0.51	0.07-3.52	0.49
R2	Reference			Reference		
Adjuvant chemotherapy	6.24	1.89-20.62	0.003	0.65	0.09-4.85	0.67
Number of lymph nodes with metastasis*^2^	1.08	1.05-1.12	< 0.001	1.13	1.04-1.23	0.003

*1: Multivariate hazard ratios (HR), 95% confidence intervals (CI), and *P *values were adjusted for age at diagnosis (years), gender, calendar month of blood sampling, cancer stage (I, II, III, and IV), residual tumor after surgery (R0, no residual tumor; R1, microscopic residual tumor; R2, macroscopic residual tumor), time period of surgery (between May 2003 and December 2005 or between January 2006 and January 2008), location of tumor, adjuvant chemotherapy, and number of lymph nodes with metastasis.

*2: Lymph nodes were resected at surgery, and metastases in lymph nodes were confirmed pathologically.

**Table 5:** Cox proportional hazard models of disease-free survival in patients with type of resection of R0 without or with multivariate adjustment*^1^

Variable	Single-variate analyses			Multivariate analysis		
		
	Crude HR	95% CI	*P *value	AHR	95% CI	*P *value
25OHD, ng/mL	1.00	0.95-1.05	0.86	1.02	0.96-1.10	0.50
Age at surgery, years	1.01	0.98-1.04	0.64	1.00	0.96-1.04	0.95
Gender, female	0.69	0.31-1.56	0.38	0.68	0.25-1.86	0.46
Time period of surgery						
May 2003 - December 2005	0.45	0.20-1.03	0.06	0.42	0.11-1.54	0.19
January 2006 - January 2008	reference					
Stage						
Stage I	0.00	-	-	0.00	-	-
Stage II	0.20	-	0.13	0.00	-	-
Stage III	0.68	0.18-0.56	0.71	0.00	0.00-0.00	< 0.001
Stage IV	Reference			Reference		
Location of tumor						
Proximal colon	1.14	0.49-2.64	0.56	0.69	0.23-2.03	0.50
Distal colon	0.43	0.12-1.55	0.20	0.57	0.13-2.40	0.44
Rectum	Reference			Reference		
Adjuvant chemotherapy	4.49	1.71-11.75	0.002	0.68	0.18-2.64	0.58
Number of lymph nodes with metastasis*^2^	1.06	1.02-1.11	0.007	1.06	0.96-1.18	0.24

*1: Multivariate hazard ratios (HR), 95% confidence intervals (CI), and *P *values were adjusted for age at diagnosis (years), gender, calendar month of blood sampling, cancer stage (I, II, III, and IV), residual tumor after surgery (R0, no residual tumor; R1, microscopic residual tumor; R2, macroscopic residual tumor), time period of surgery (between May 2003 and December 2005 or between January 2006 and January 2008), location of tumor, adjuvant chemotherapy, and number of lymph nodes with metastasis.

*2: Lymph nodes were resected at surgery, and metastases in lymph nodes were confirmed pathologically.

## Discussion

In this study, the median serum 25OHD levels in patients with colorectal cancer was 10 ng/mL, and 87% were in a vitamin D-insufficient state (less than 30 ng/mL), which is lower than expected. SRL Inc. measured 25OHD, which has a range of 7 ng/mL to 41 ng/mL as determined by data from healthy volunteers [19]. When data from the US National Health and Nutrition Examination Survey (NHANES) 2001-2004 were compared with data from 1988-1994, the prevalence of 25OHD levels less than 10 ng/mL increased from 2% to 6% in total and 9% to 29% in non-Hispanic blacks, with a corresponding decrease in the prevalence of levels of 30 ng/mL or more from 45% to 23% and 12% to 3%, respectively [21]. The population of this study was Japanese, and the average skin color was between that of white and black subjects. The study period was between 2003 and 2008, which, although partly overlapping the later period of NHANES 2001-2004, was conducted mostly after that study. Moreover, populations who develop colorectal cancer tend to have lower serum 25OHD levels [5,6]. Thus, a higher prevalence of 25OHD insufficiency in Japanese patients with colorectal cancer after 2003 may be plausible, although we do not have exact controls to verify this hypothesis.

Levels of 25OHD oscillated with the seasons, showing lower levels in spring and higher levels in late summer. This finding was similar to that reported in a British study [22]. In an ecological study, the survival of colon cancer patients in Norway was highest for those diagnosed in the summer and autumn [23]. These findings are consistent with our results.

Among 257 Japanese patients with colorectal cancer, higher 25OHD levels were associated with a significantly better overall survival under Cox proportional hazard models with multi-variate adjustment for month of serum sampling as well as age at diagnosis, gender, cancer stage, residual tumor after surgery, time period of surgery, and number of lymph nodes with metastasis at surgery, which is consistent with previous studies [14,15]. However, without adjustment, there was no association between 25OHD levels and overall survival. There was no significant association between 25OHD levels and colorectal cancer-specific deaths or disease-free survival. A meta-analysis showed that vitamin D supplements may improve overall survival [24] as well as cardiovascular mortality [25]. Thus, patients with higher vitamin D levels may have an advantage in both cancer- and non-cancer-related deaths, leading to an increase in overall survival rather than disease-free survival.

In patients with early-stage non-small-cell lung cancer [26] or breast cancer [27], higher 25OHD levels were reported to be associated with better prognosis. In contrast, Freedman et al. examined 536 cancer deaths in 146,578 person-years, and found an inverse relationship between 25OHD levels and colorectal cancer mortality, but not other types of cancers [28]. A positive association between serum 25OHD levels and survival may be observed in colorectal cancer, but is still controversial in other types of cancers.

This study has several limitations. Our study population was not large enough to detect minor differences in 25OHD levels. Moreover, our findings could be limited because blood samples were taken only at the time of surgery and not at diagnosis; it is possible that samples taken at the time of diagnosis may have led to different results, because lifestyle factors, including outdoor activities and body mass index which are strongly associated with 25OHD levels might change in patients, when they are told they have colorectal cancer. However, because we did not include patients treated with chemotherapy and/or radiotherapy before surgery, we felt that changes in serum 25OHD levels from diagnosis to time of surgery would not be big. In addition, after surgery, 25OHD levels may change because of changes in physical activity, body mass index, and diet. However, because we only took blood samples at the time of surgery, we could not examine these potential effects. Moreover, we did not measure possible confounders such as physical activity and body mass index. In addition, because this was an observational study, treatment including adjuvant chemotherapy was not standardized, as it would have been in a clinical trial setting. Thus, the choice of therapy could also have an effect on the overall survival. Jikei University Hospital is a private hospital, and most patients were thought to live in Tokyo and the surrounding prefectures. Thus, our results could also have been confounded by socioeconomic factors. For example, patients living in Tokyo area may have higher opportunity to be diagnosed with colorectal cancer at an earlier stage because they are more likely to undergo screening tests or a medical check up, which can be associated with better survival. On the other hand, they may spend more time indoors, which could help explain why serum 25OHD levels were low. Recently, single nuclear polymorphisms of the vitamin D receptor were reported to strongly affect survival among patients with non-small-cell lung cancer [29]. Synergistic effects of single nuclear polymorphisms of vitamin D receptor and 25OHD on survival should be prospectively studied in the future. Moreover, clinical trials using high doses of vitamin D supplements are warranted to determine if vitamin D truly improves survival of patients with colorectal cancer.

## Conclusions

These results suggest that higher 25OHD levels at surgery may be associated with a better survival rate of patients with colorectal cancer.

## Competing interests

The authors declare that they have no competing interests.

## Authors' contributions

Substantial contributions to conception and design: TS, MW, MU

Acquisition of data: HM, TS, MW, KY

Analysis and interpretation of data: HM, TS, CN, DT, AS, ST, YT, MU

Drafting manuscript: HM, MU

Revising manuscript critically for important intellectual content: TS, MW

Final approval of the version to be published: All the authors

## Pre-publication history

The pre-publication history for this paper can be accessed here:

http://www.biomedcentral.com/1471-2407/10/347/prepub
